# Supplementary material for: Force generation of KIF1C is impaired by pathogenic mutations
Source: Curr Biol. 2022 Sep 12;32(17):3862–3870.e6. doi: 10.1016/j.cub.2022.07.029 (PMC9631238; doi:10.1016/j.cub.2022.07.029)
Supplement: Document S1. Figures S1 and S2 [file mmc1.pdf]

Current Biology, Volume *32*

Supplemental Information

Force generation of KIF1C is impaired

by pathogenic mutations

Nida Siddiqui, Daniel Roth, Algirdas Toleikis, Alexander J. Zwetsloot, Robert A. Cross, and Anne Straube

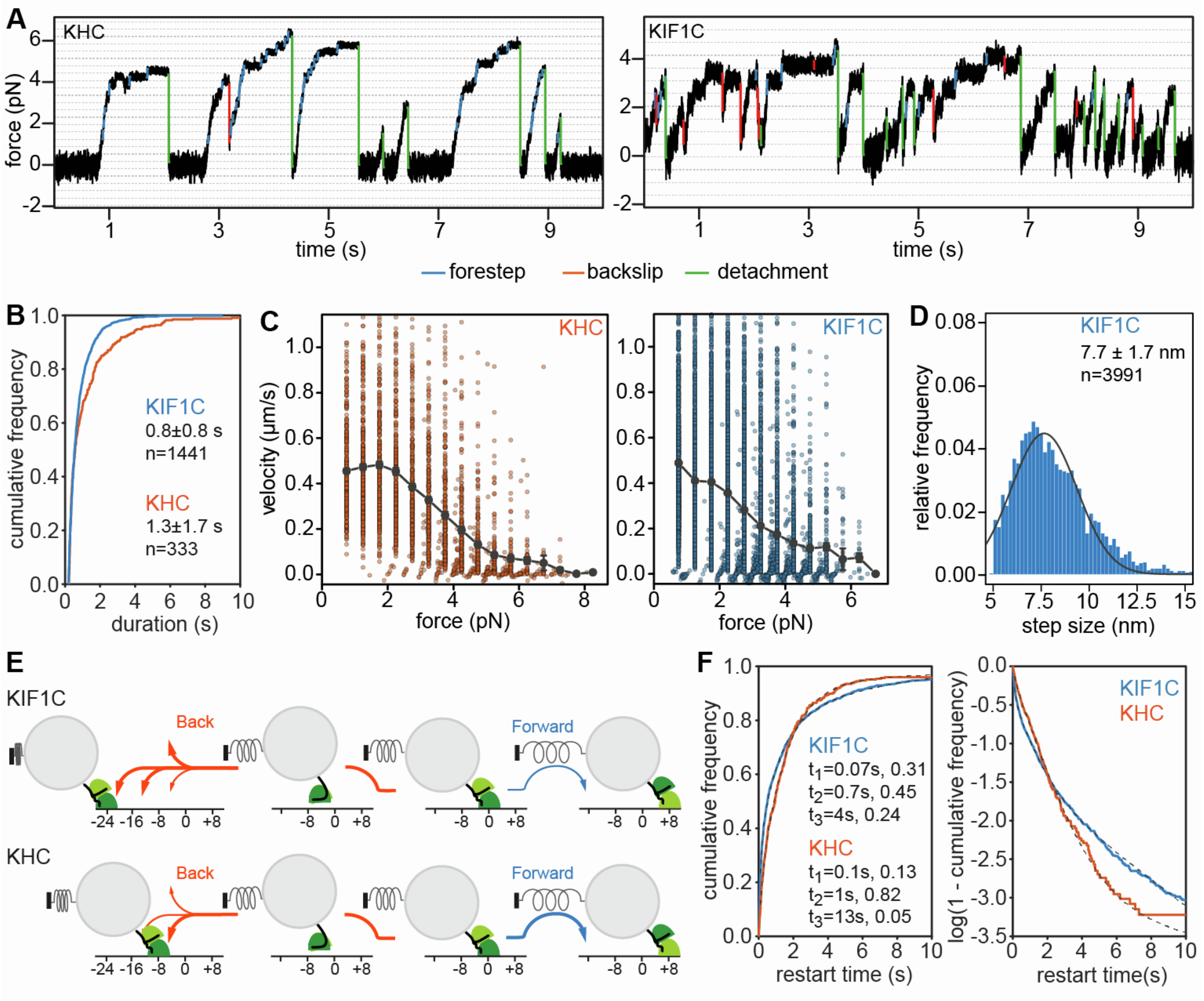

Figure S1: Step and force event analysis, related to Figure 1.

(A) Representative traces from single bead optical trapping experiment for Drosophila melanogaster kinesin heavy chain (KHC) and human KIF1C. Dotted lines indicate 8 nm displacements. The steps detected using the step-finder are highlighted. Forward steps in blue, backslips in red and detachments in green. Parameters used in step finder algorithm are t-test score threshold=30, minimum step size=5 nm, minimum force=1 pN, moving average=20 (1 ms). **(B)** Cumulative distribution of durations over which a force of at least 0.5 pN was maintained, mean ± SD shown, p=0.0002 (Kolmogorov-Smirnov test). **(C)** Distribution of velocity of KHC and KIF1C relative to force. n=353 for KHC and n=1662 for KIF1C. **(D)** Forward step-size distribution for KIF1C. Gaussian curve fit in grey with μ = 7.7 nm, σ= 1.7 nm, n=3991. **(E)** Model highlighting differences in backslipping and restarting probabilities for KIF1C and KHC. KIF1C is more likely to backslip under force and backslips tend to be longer. **(F)** Linear and log plots of cumulative distribution of time lag between force generating events, i.e. time until 0.5 pN force was generated following a backslip or detachment that resulted in force to be reduced below 0.5 pN. n=326 for KHC and n=1404 for KIF1C. Triexponential fits are shown as grey dashed lines and half-times as well as fractions for each mode are indicated in the legend. $p=6 \cdot 10^{-6}$ (Kolmogorov-Smirnov test). See also Methods S1.

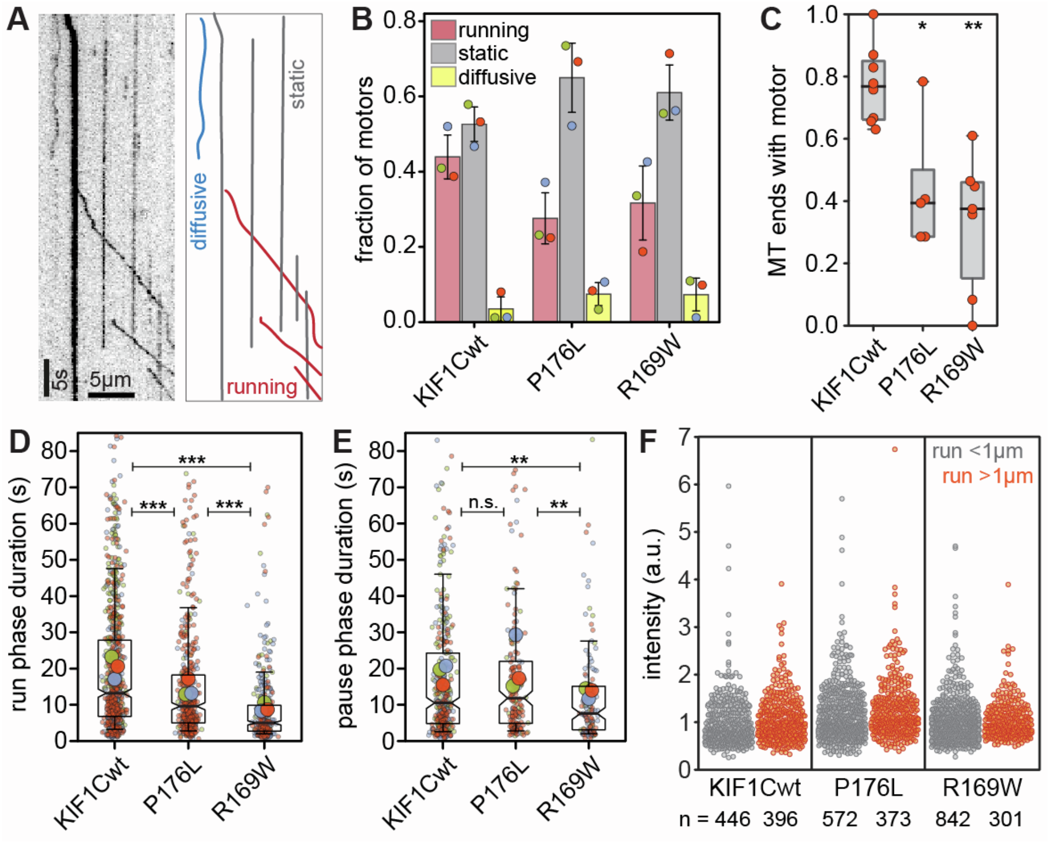

Figure S2: Single molecule behaviour of KIF1C with pathogenic mutations, related to Figure 2.

(A) Three types of behaviour were observed in kymographs: static motors (grey traces), diffusing motors (blue trace) and running motors (red traces). **(B)** Fraction of motors that either run towards the plus end, are static or diffusive. Bars show mean ± SD of 3 experiments, and coloured dots indicate individual values for each experiment. Data from the 3 groups are not statistically different at 0.05 cutoff. (Kruskal Wallis, Conover's test post hoc with Holm correction). **(C)** Fraction of microtubules with at least one motor decorating one end. This was determined at a timepoint about 3 minutes after start of the motility assay, i.e. in the first frame of the second movie in each chamber, by manual assessment. Box plot with data overlay shows median, quartiles and min/max values (whiskers). n = 8, 5, 7 chambers, respectively from 3 independent experiments. * p=0.0164; ** $p=3 \cdot 10^{-4}$ (Kolmogorov-Smirnow test). **(D-E)** Superplots for phase durations for plus end-directed runs and pauses of individual motors (small dots), averages per experiment day (large dots) are shown colour-coded by experiment day. Boxes indicate quartiles and whiskers at 10/90th percentiles. Note that values outside of the Y axis limits have been omitted from the graph, but are included in the statistics. n=853, 463 and 384 run phases and 404, 178 and 131 pause phases from 3 experiments. n.s. p=0.52; ** p<0.005; *** p<0.0001 (Kruskal Wallis, Conover's test post hoc with Holm correction). **(F)** Intensity of motors analysed in motility assays separated into a highly processive group with run length > 1 µm (orange) and a group that were either static or underwent a short run < 1 µm (grey). The majority of motors fall into the main peak area of presumably dimers. Higher order motor clusters contribute to a comparable extent to both groups. Number of motors analysed is indicated for each group under the graph. Data were pooled from two independent experiments.
