# Supplementary material for: Force generation of KIF1C is impaired by pathogenic mutations
Source: Curr Biol. 2022 Sep 12;32(17):3862–3870.e6. doi: 10.1016/j.cub.2022.07.029 (PMC9631238; doi:10.1016/j.cub.2022.07.029)
Supplement: Document S2. Article plus supplemental information [file mmc3.pdf]

Report

Current Biology

Force generation of KIF1C is impaired by pathogenic mutations

Highlights

- KIF1C stalls at loads of ~3.7 pN, which is intermediate between kinesin-1 and KIF1A
- KIF1C has a propensity to backslip while remaining weakly attached to the microtubule
- HSP-causing mutations in KIF1C retain fast, processive single-molecule motility
- KIF1C mutations are severely impaired under the load of an optical trap or cellular cargo

Authors

Nida Siddiqui, Daniel Roth, Algirdas Toleikis, Alexander J. Zwetsloot, Robert A. Cross, Anne Straube

Correspondence

anne@mechanochemistry.org

In brief

Siddiqui et al. characterize single-molecule force generation by kinesin-3 KIF1C and report the intermediate properties between the strong kinesin-1 and backslip-prone kinesin-3 KIF1A. Two KIF1C mutations causing hereditary spastic paraplegia permit fast and processive single-molecule motility but are severely impaired under load.

Siddiqui et al., 2022, Current Biology *32*, 3862–3870
September 12, 2022 © 2022 The Author(s). Published by Elsevier Inc.
https://doi.org/10.1016/j.cub.2022.07.029

CellPress

CellPress
OPEN ACCESS

Current Biology

Report

Force generation of KIF1C is impaired by pathogenic mutations

Nida Siddiqui,[1,2] Daniel Roth,[1] Algirdas Toleikis,[1,3] Alexander J. Zwetsloot,[1,4] Robert A. Cross,[1] and Anne Straube[1,5,6,*]
[1]Centre for Mechanochemical Cell Biology, Division of Biomedical Sciences, Warwick Medical School, University of Warwick, Coventry CV4 7AL, UK
[2]Present address: AstraZeneca, Granta Park, Cambridge CB21 6GH, UK
[3]Present address: Vilnius University, Life Sciences Center, 7 Saulėtekio Ave, 10257 Vilnius, Lithuania
[4]Present address: GlaxoSmithKline, Gunnels Wood Road, Stevenage SG1 2NY, UK
[5]Twitter: @AnneStraube
[6]Lead contact
*Correspondence: anne@mechanochemistry.org
https://doi.org/10.1016/j.cub.2022.07.029

SUMMARY

Intracellular transport is essential for neuronal function and survival. The most effective plus-end-directed neuronal transporter is the kinesin-3 KIF1C, which transports large secretory vesicles and endosomes.[1–4] Mutations in KIF1C cause hereditary spastic paraplegia and cerebellar dysfunction in human patients.[5–8] In contrast to other kinesin-3s, KIF1C is a stable dimer and a highly processive motor in its native state.[9,10] Here, we establish a baseline for the single-molecule mechanics of Kif1C. We show that full-length KIF1C molecules can processively step against the load of an optical trap and reach average stall forces of 3.7 pN. Compared with kinesin-1, KIF1C has a higher propensity to slip backward under load, which results in a lower maximal single-molecule force. However, KIF1C remains attached to the microtubule while slipping backward and re-engages quickly, consistent with its super processivity. Two pathogenic mutations, P176L and R169W, that cause hereditary spastic paraplegia in humans[7,8] maintain fast, processive single-molecule motility *in vitro* but with decreased run length and slightly increased unloaded velocity compared with the wild-type motor. Under load in an optical trap, force generation by these mutants is severely reduced. In cells, the same mutants are impaired in producing sufficient force to efficiently relocate organelles. Our results show how its mechanics supports KIF1C's role as an intracellular transporter and explain how pathogenic mutations at the microtubule-binding interface of KIF1C impair the cellular function of these long-distance transporters and result in neuronal disease.

RESULTS AND DISCUSSION

Force generation properties of KIF1C

To determine the force output of KIF1C, we used single-bead optical tweezer assays,[11,12] full-length human KIF1C,[10] and, for comparison, full-length *Drosophila melanogaster* kinesin heavy chain (KHC).[13,14] Forces were calculated from bead displacements under conditions in which less than 20% of beads move, thus ensuring that a single kinesin pulls on the bead. Examining the force traces suggested that KIF1C and KHC produced force events of comparable magnitude and duration, although KIF1C events were disrupted twice as frequently by long backward slips (Figures 1A, 1B, and S1). The median duration of force events was about 0.5 s for both motors, but KHC showed a significantly larger fraction of force events lasting longer than 2 s (Figure S1). The average stall force of KHC, i.e., the force that motors held for at least 10 ms before the bead returned to the trap center, was 5.4 pN, in agreement with the literature.[15–17] KIF1C forces were ∼30% lower and stalled at 3.7 pN on average. Stall forces were consistent over a range of minimal stall-time criteria, from 10 to 800 ms, with the average stall time being 0.3 s for KIF1C and 0.5 s for KHC (Figures 1C–1E). Free-running beads with a single KIF1C moved at 922 ± 40 nm/s, but their average forward speed rapidly decreased under the load of the trap and reached zero at 7 pN. Although KHC's unloaded velocity was ∼20% slower, KHC moved at significantly higher speeds at forces >1 pN, maintaining an average speed of 450 nm/s at forces <2.5 pN before slowing down rapidly, with forward speed reaching zero at 8 pN (Figures 1F and S1). To look at the force-dependence of the motors more closely, we analyzed forward and backward steps. As expected, KIF1C took forward steps of approximately 8 nm in length (Figure S1), consistent with all other kinesins studied so far.[12,18–22] The force at which the probability of taking a forward step or slipping backward is equal, was 7.3 pN for KHC and 5.7 pN for KIF1C (Figure 1G). These values are consistent with the ∼98th percentile of stall forces we measured (Figures 1C and 1D). Closer inspection of the step sizes observed at different forces (Figure 1H) revealed that KHC backslips once every 9 detectable forward steps, and the vast majority of KHC backward displacements are 8 nm. Kif1C backslips twice as frequently, and 3 times as many KIF1C backslips are larger than 12 nm, suggesting that

 © 2022 The Author(s). Published by Elsevier Inc.
This is an open access article under the CC BY license (http://creativecommons.org/licenses/by/4.0/).

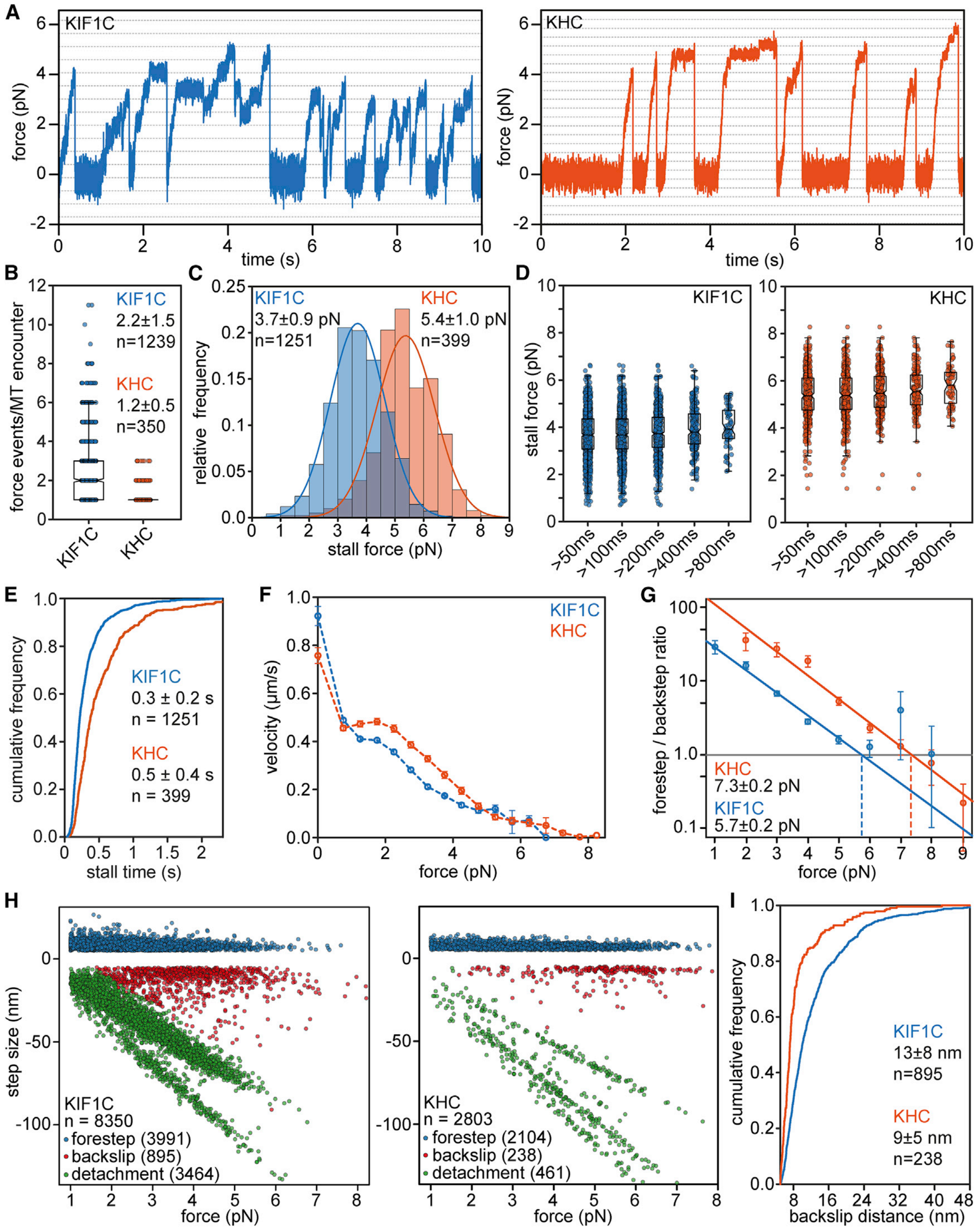

Figure 1. KIF1C single-molecule force generation

(A) Representative traces from single-bead optical trapping experiment for human KIF1C and *Drosophila melanogaster* kinesin heavy chain (KHC). Dotted lines indicate 8-nm displacement.

(B) Number of force-producing events per microtubule (MT) encounter for KIF1C and KHC. Boxes indicate quartiles and whiskers at 10/90th percentile. Data shown are mean ± SD. n = 350, 1,239 MT encounters analyzed. $p = 1\times10^{-5}$ (Kolmogorov-Smirnov test).

(C) Distributions of stall forces held for a minimum of 10 ms by KIF1C and KHC. Average force ± SD of Gaussian fit shown. n = 399, 1,251 stall events analyzed. $p = 5\times10^{-160}$ two-sample t test.

(D) Stall force distributions as a function of minimal stall time for KIF1C and KHC. Individual force events overlaid by box plot indicating quartiles and whiskers at 10/90th percentile. For KIF1C: 50 ms: 3.69 ± 0.03 pN, n = 1,244; 100 ms: 3.69 ± 0.03 pN, n = 1,177; 200 ms: 3.77 ± 0.04 pN, n = 715; 400 ms: 3.91 ± 0.06 pN, n = 263; 800 ms: 4.0 ± 0.1 pN, n = 70. For KHC: 50 ms: 5.37 ± 0.05 pN, n = 398; 100 ms: 5.39 ± 0.05 pN, n = 392; 200 ms: 5.53 ± 0.005 pN, n = 326; 400 ms: 5.60 ± 0.07 pN, n = 187; 800 ms: 5.81 ± 0.1 pN, n = 68. Data listed are mean ± SEM.

(legend continued on next page)

KIF1C is both more likely to slip backward and to slip further when it does so (Figures 1H and 1I). Indeed, a significant fraction of KIF1C detachment events—defined as backward motion whereby the bead returns to the trap center—might be long backward slips whereby the motor remains (weakly) attached to the microtubule. The time lag between detachments and the start of a new force-generating event follows a triexponential distribution for both motors (Figure S1). The fastest timescale presumably reflects long backslips where the force reduces below the 1.2 pN cutoff. This accounts for 31% of KIF1C detachment events ($t_{½}$ = 74 ms) and 13% of KHC detachments ($t_{½}$ = 104 ms) (Figure S1). After reassigning these events, KIF1C has a 3.5-fold increased propensity to backslip and a slightly higher ratio of backslips versus detachments than KHC.

Taken together, single molecules of full-length human KIF1C can generate substantial stall forces, although their force output is about 30% lower than that of conventional kinesin. For comparison, the average stall forces of constitutively active KIF1A and Unc104 were reported as 3.0 and 2.6 pN, respectively,[17] while monomeric truncated and native full-length KIF1A are only weakly processive, with a stall force below 0.3 pN.[17,20] In addition to significantly higher stall forces compared with constitutively active KIF1A, KIF1C also stalls for significantly longer. Most KIF1C stalls last several 100 ms, while only 20% of hyperactive KIF1A stalls exceed 50 ms.[17] While KIF1C has a 3.5-fold increased probability of backward slipping under load compared with KHC, constitutively active KIF1A shows 50-fold enhanced slipping.[17,23] Thus, our data suggest that KIF1C has unique force-generating properties that are intermediate between KIF1A and kinesin-1. These intermediate properties might enable KIF1C to be both highly processive[10] and generate substantial forces at low motor numbers, which might explain why KIF1C has been identified as the most effective cellular cargo transporter.[1]

We proposed recently that kinesin-1 backslips arise when phosphate is released before a forward step is completed, which converts the motor into a weakly bound state that is pulled backward under the load of the trap. Backward slipping is stopped when one of the two motor domains in the ADP state re-engages at a binding site and releases nucleotides to start another cycle of trying to make a forward step.[14] This proposed pathway was supported by recent high bandwidth trapping data, indicating continuous engagement of kinesin-1 with the microtubule during backward slips.[24] At zero load, kinesin-3s are about 10-fold more processive and twice as fast as kinesin-1.[25,26] The observations that KIF1C and KIF1A undertake backslips more frequently suggests that they release phosphate faster, which might be a trade-off for being faster motors. Consistent with this, KIF1A was proposed to exist in a one-head-bound or weakly bound state for more than 90% of its mechanochemical cycle.[27] However, due to their interaction with microtubules being significantly stronger,[26] kinesin-3s stay attached to the microtubule even in a weakly bound state for long enough to wait for the next step. The ability to slip in a weakly bound state might also help kinesin-3 motors to work efficiently in teams, as it might permit motors to slip forward as well as backward, allowing the team to potentially move faster than a single motor.[28]

Pathogenic mutations in KIF1C increase off rate and unloaded speed

In recent years, disease-causing mutations in KIF1C have been identified in patients presenting with complex forms of hereditary spastic paraplegia or spastic ataxias. Two of these, P176L[7] and R169W,[8] are located in the motor domain of KIF1C at the microtubule-binding interface (Figure 2A), but how these affect the biophysical properties of KIF1C is currently unclear. Thus, we introduced both mutations and purified full-length recombinant human KIF1C-GFP from insect cells (Figure 2B). To investigate the effect of these pathogenic mutations, we first determined in single-molecule assays whether the motors can still bind and move along microtubules. Both, $KIF1C_{P176L}$-GFP and $KIF1C_{R169W}$-GFP were motile in the single-molecule motility assays and could still reach the microtubule plus ends (Figures 2C and 2D), suggesting that the mutants are still able to move processively along microtubules. The landing rate of all motors was comparable, suggesting that the mutations do not alter the balance of autoinhibited versus active motors nor the initial binding occurring in the ADP state (Figure 2E). Under the conditions of the assay, we observe slightly more than half of the motors being static or moving very slowly (< 25 nm/s). The fraction of static motors was slightly increased for both mutants. Diffusing motors are observed occasionally for wild-type (WT) KIF1C but were increased more than 2-fold for the mutants. This resulted in a ~30% reduction in motile motors (Figure S2). We also observed a 50% reduction in the fraction of microtubule plus ends decorated by a kinesin for each of the mutants (Figure S2). Detailed analysis of single-molecule motility revealed that the dwell time of both mutants was significantly reduced (Figure 2F). As both mutations are located at the microtubule-binding interface of KIF1C, the increase in diffusive events and faster off rates suggest that microtubule binding of both mutants is reduced. Even though the speed was increased (Figure 2G), the fast off rate resulted in a significantly shorter run length of both mutants compared with WT KIF1C (Figure 2H). KIF1C motors frequently

(E) Cumulative distribution of durations over which a force of at least 0.5 pN was maintained. Data shown are mean ± SD. n = 1,251, 399 force events. p = 0.0002 (Kolmogorov-Smirnov test).
(F) Force-velocity relationship for KIF1C (analyzed from n = 1,662 individual runs) and KHC (analyzed from n = 353 individual runs). Unloaded velocities were determined from videos of beads after switching the trap off. n = 16 runs for KIF1C, and n = 26 runs for KHC. Data shown are mean ± SEM.
(G) Forestep to backstep ratio relative to force shows that KIF1C reaches fore/backstep balance at 5.7 ± 0.2 pN and KHC at 7.3 ± 0.2 pN. Error from linear regression fit. Note that superstall events obtained under added resistive loads are only included in the KHC dataset. n = 2,686 steps for KHC and 4,602 steps for KIF1C.
(H) Step sizes extracted from single-molecule force recordings of KIF1C and KHC plotted relative to the force at which they originated. Number of events for each category are given in brackets.
(I) Cumulative distribution of backslip distances. n = 238, 895 backslips analyzed. Data shown are mean ± SD. $p = 1 \times 10^{-22}$ (Kolmogorov-Smirnov test).
See also Figure S1 and Methods S1.

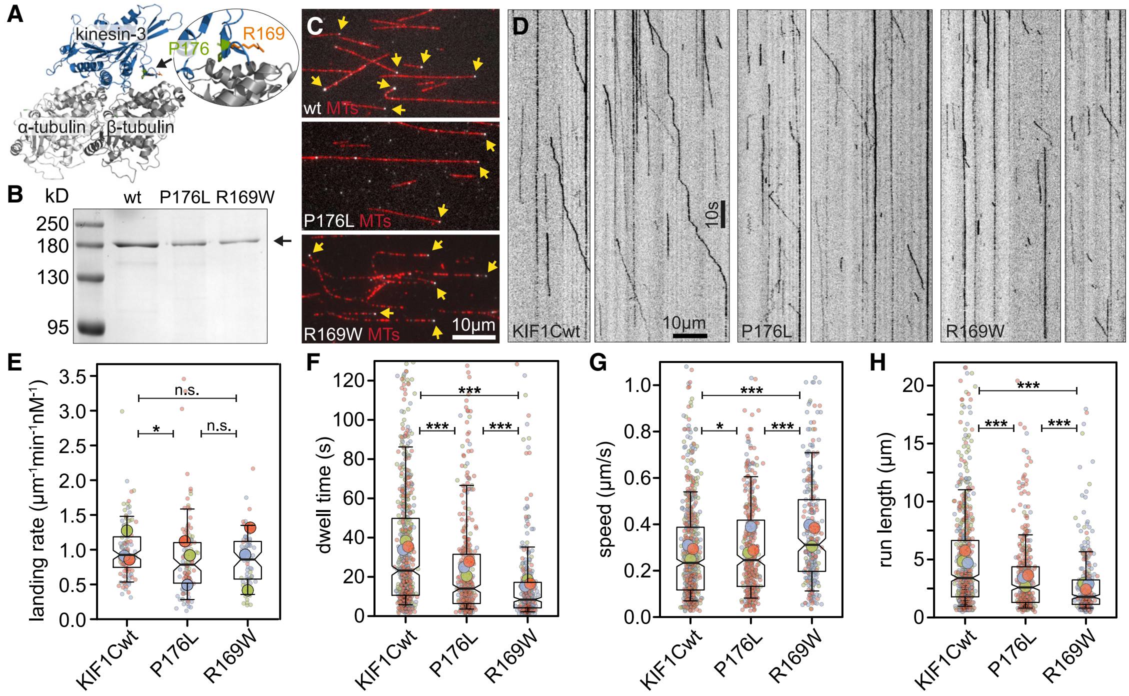

Figure 2. Pathogenic mutations in KIF1C increase unbinding rate and speed

(A) Structure of a kinesin-3 motor domain (blue) bound to a microtubule (gray), with the residues R169 and P176 highlighted. Structure is of related motor KIF1A (PDB: 4UXP) in which both residues are conserved.

(B) SDS PAGE analysis of purified recombinant human KIF1C-GFP wild type, KIF1C$_{P176L}$-GFP, and KIF1C$_{R169W}$-GFP, and molecular size markers as indicated.

(C) TIRF micrograph of Taxol-stabilized microtubules (red) and full-length recombinant human KIF1C tagged with GFP (wild type or carrying mutations as indicated) in grayscale. Plus-end accumulation is indicated with yellow arrows.

(D) Representative kymographs showing motility of KIF1C-GFP wild type and pathogenic mutants P176L and R169W.

(E) Superplot showing the landing rate of motors for each microtubule analyzed (small dots) and per experiment day (large dots) are color-coded by experiment day. Boxes indicate quartiles and whiskers at 10/90th percentile. n = 114, 109, and 65 microtubules from 3 experiments. n.s. $p > 0.05$; *$p < 0.05$; (Kruskal-Wallis test, Conover's test post hoc with Holm correction).

(F–H) Superplots for dwell time, average (pause-corrected) speed and run length of individual motors (small dots), averages per experiment day (large dots) are shown color-coded by experiment day. Boxes indicate quartiles and whiskers at 10/90th percentile. Note that values outside of the y axis limits have been omitted from the graph but are included in the statistics. n = 697, 409, and 305 motors from 3 experiments. *$p < 0.05$; ***$p < 0.0001$ (Kruskal-Wallis test, Conover's test post hoc with Holm correction).

See also Figure S2.

pause during runs (Figure 2D); the duration of run phases was more strongly reduced as a result of the mutations than the duration of pauses (Figure S2), suggesting that motors are more prone to detachment during runs. Thus, our single-molecule analysis of full-length KIF1C WT and P176L and R169W mutants suggests that both pathogenic mutations reduce microtubule-binding affinity, which results in faster motility but a higher off rate and shorter run length.

The two characterized mutations are both located in loop8/strand-β5, which was shown to make additional connections to the microtubule surface in kinesin-3 motors compared with kinesin-1.[29] When two mutations were simultaneously introduced into this region in KIF1A (R167S and H171D), the run length was 10-fold and the speed 2-fold reduced, matching kinesin-1 motility properties.[26] Thus, our findings that the two pathogenic mutations in KIF1C retain motility but with reduced run length is consistent with this. Pathogenic mutations in other regions of the microtubule-binding interface of KIF1A[23,30] also showed reduced run lengths. However, we found that the unloaded velocity of the motors was not negatively affected and even slightly increased, while mutations across the microtubule-binding interface of KIF1A resulted in lower single-molecule velocities.[23,26,30] Alanine scanning mutagenesis in kinesin-1 also identified two mutations (E220A and L317A) with a slightly higher velocity but reduced microtubule affinity.[31] Thus, the different outcome of reducing microtubule affinity on unloaded speed across kinesin-3 motors could be due to the biophysical properties of KIF1C being intermediate between kinesin-1 and KIF1A.

Pathogenic mutations in KIF1C severely reduce force generation

Molecular motors haul cargoes against the viscous drag of the cytoplasm and various intracellular structures. Thus, to understand how the reduced microtubule affinity affects the ability to step under load, we analyzed both KIF1C$_{P176L}$-GFP and KIF1C$_{R169W}$-GFP using the optical trap. At the trap stiffness we used to analyze KIF1C WT motors (Figure 1), we did not observe

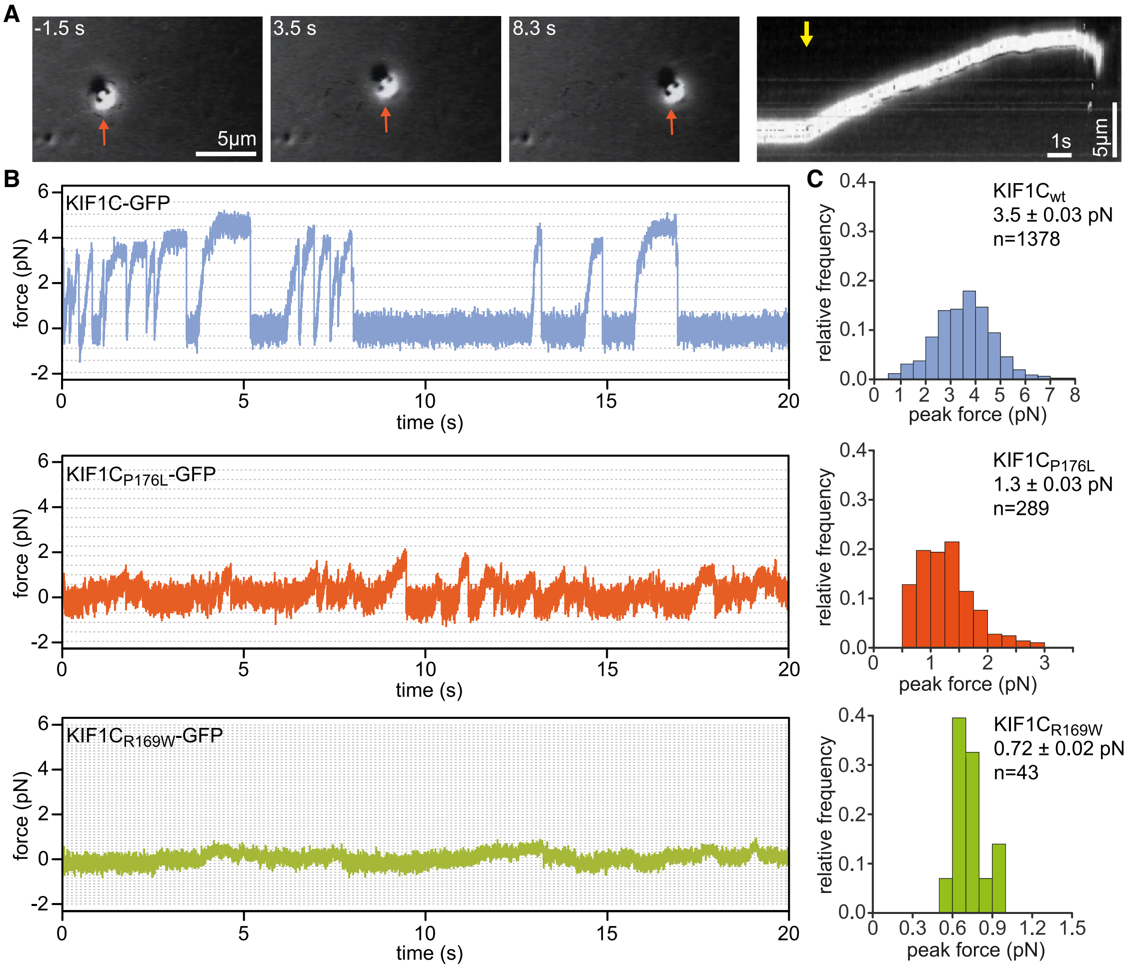

Figure 3. Pathogenic mutations of KIF1C impair force generation

(A) Stills and kymograph from an optical trapping experiment showing processive bead transport by KIF1C$_{P176L}$-GFP after release of the motor from the trap (bead indicated with orange arrow in stills, indicated time relative to trap release indicated by yellow arrow in kymograph).

(B) Representative traces from optical trapping experiment with KIF1C-GFP wild type and pathogenic mutants P176L and R169W. Dashed lines indicate 8-nm displacement intervals, which are narrower at the low trap stiffness used for mutants.

(C) Histograms of peak forces determined for events lasting longer than 200 ms that exceed 0.5 pN. Mean ± SEM and number of force events are indicated. Groups are significantly different with $p < 0.0001$ (Kruskal-Wallis test, with Sidak correction).

significant bead displacements for the mutants. To confirm that motors were present on the beads, we positioned beads on the microtubule and then switched the trap off to let the motor run without load. This confirmed the presence of functional motors (Figure 3A), but these were too weak to generate forces comparable to the WT motor. We then collected bead displacement data at reduced trap stiffness (see STAR Methods for details) and detected short runs for KIF1C$_{P176L}$-GFP with an average peak force of 1.3 pN, compared with 3.5 pN for the WT motor (Figures 3B and 3C). KIF1C$_{R169W}$-GFP moved the bead only small distances when the trap was switched off and could not generate forces above 1 pN (Figures 3B and 3C). These data suggest that the moderate reduction in processivity of unloaded motors observed in single-molecule assays are exacerbated once the motors generate forces against a load.

Thus, P176L and R169W affect KIF1C force generation much more dramatically than their unloaded motility. This seems not to be the case for the recently characterized P305L mutation in the 3_{10}-helix of KIF1A, which showed a roughly 5-fold reduced landing rate, a 2-fold reduced speed and run length, and a 2-fold reduced force.[23] It will require detailed analysis of mutations in different regions of the microtubule-binding interface to understand whether these differences are due to a specific function of the loop8/strand-β5 region in limiting the velocity and enhancing force generation of KIF1C or whether the relationship of microtubule affinity and force generation differs between motors from the kinesin-3 family.

Pathogenic mutations impair cellular cargo transport

In cells, motors could work in teams and thereby compensate for reduced single-molecule force. To test this, we first analyzed the ability of KIF1C with pathogenic mutations to localize to the cell periphery, which would indicate their ability to move through the cytoplasm.

CellPress
OPEN ACCESS

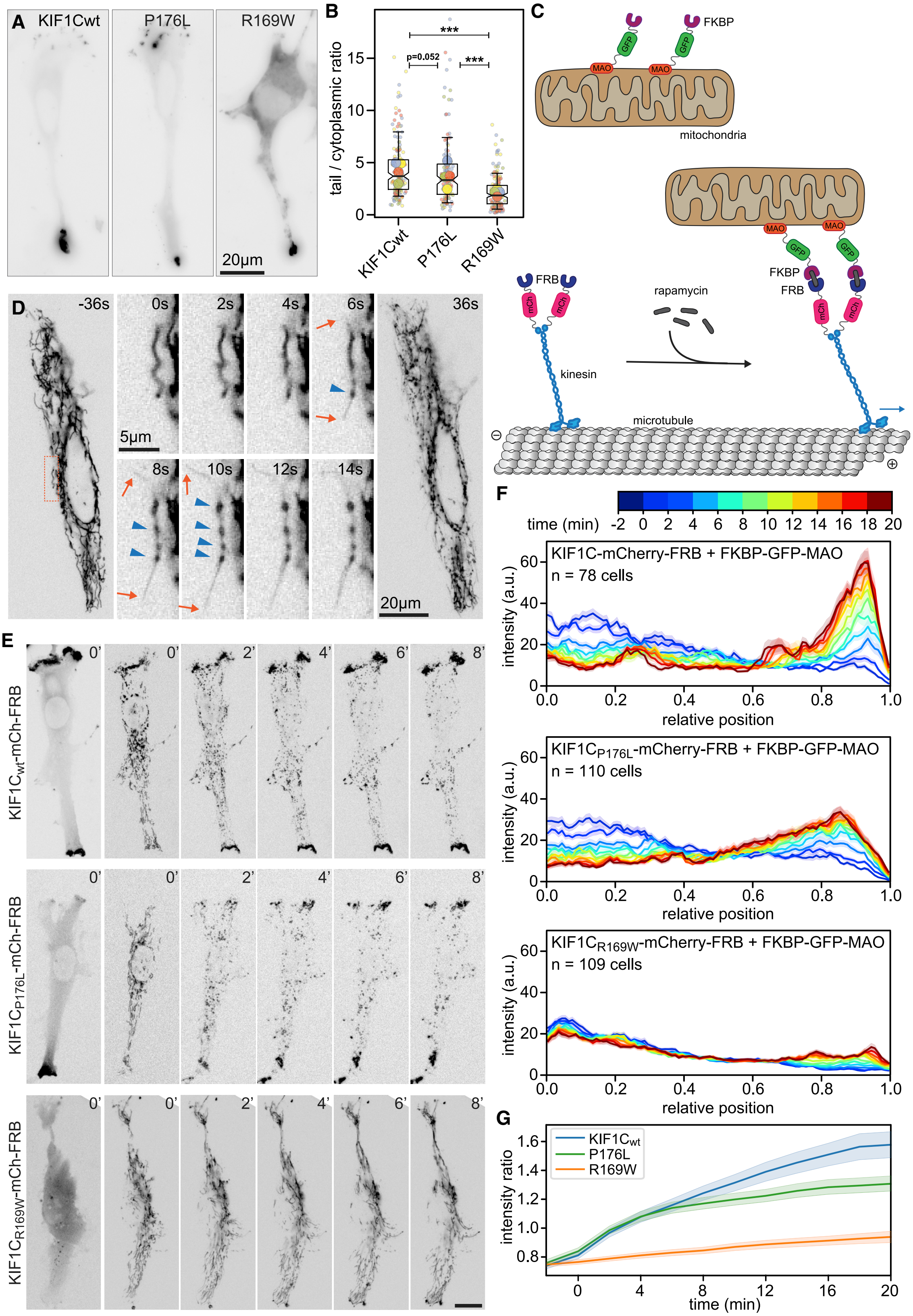

(legend on next page)

KIF1C-GFP accumulates in the tails of migrating RPE1 cells as previously reported.[4,10] $KIF1C_{P176L}$-GFP and $KIF1C_{R169W}$-GFP were also found in cell tails, but comparable tail accumulation was only reached at higher cytoplasmic levels (Figure 4A). Quantifying the ratio of tail to perinuclear intensity revealed that tail accumulation of $KIF1C_{R169W}$-GFP was 2-fold reduced compared with WT KIF1C-GFP, while $KIF1C_{P176L}$-GFP accumulation was only very slightly reduced (Figure 4B). This suggests that single-molecule force generation above 2 pN is not required for motor relocation, but it is not clear from this assay whether tip-localized motors actively transported cargo.

To more directly test force generation in cells, we recruited WT and mutated motors to mitochondria as a large cargo expected to generate a significant amount of viscous drag but also to allow large teams of motors to work together. To do this, we co-expressed FKBP-GFP-myc-tagged monoamine oxidase (MAO) with either KIF1C-mCherry-FRB, $KIF1C_{P176L}$-mCherry-FRB, or $KIF1C_{R169W}$-mCherry-FRB. GFP-tagged MAO indicated normal mitochondrial morphology and distribution before recruiting the full-length motors to the outer mitochondrial membrane (Figure 4C). Recruitment of WT KIF1C caused dramatic changes in mitochondrial morphology within seconds of adding rapamycin. The pulling forces fragmented mitochondria into thin outer membrane tubules and beads (Figure 4D). Both tubules and beads were transported toward the cell edges efficiently, resulting in a near complete accumulation of mitochondria to the cell tips within the 20 min of the experiment (Figures 4E and 4F). $KIF1C_{P176L}$-mCherry-FRB caused the same mitochondrial beading effect, albeit more slowly, and transported mitochondria to the cell periphery less efficiently than the WT motor (Figures 4E and 4F). $KIF1C_{R169W}$-mCherry-FRB was recruited very efficiently to mitochondrial membranes and accumulated on the periphery-facing tip of the membrane. Beading was only observed in cells expressing a relatively large amount of $KIF1C_{R169W}$ and then the motor was unable to efficiently move the large (700- to 1,000-nm diameter) cargo. In most cases, normal tubular mitochondrial morphology was retained and a subset of these was transported toward the cell periphery (Figures 4E and 4F). At the beginning of the experiment, the fluorescence signal for the mitochondrial marker near the cell periphery was about 25% lower than in the perinuclear area and equal among all three conditions (Figure 4G). Immediately after adding rapamycin, the signal shifted toward the periphery, albeit at a significantly higher rate in cells expressing WT KIF1C-mCherry-FRB or $KIF1C_{P176L}$-mCherry-FRB than the R169W mutant. 20 min after adding rapamycin, the ratio of peripheral versus centrally located mitochondria was clearly lower compared with WT KIF1C (Figure 4G) (WT: 1.58 ± 0.09; P176L: 1.31 ± 0.05, $p = 0.01$; R169W: 0.94 ± 0.04, $p = 3 \times 10^{-11}$ [mean ± SEM, t tests with Bonferroni correction]). Thus, pathogenic mutations in KIF1C resulting in reduced single-molecule force also have a reduced ability to relocate cargoes in cells, even when working in teams.

Our work suggests that KIF1C has biophysical properties that are intermediate between kinesin-1 and KIF1A with regard to the maximal force generated, stall times, and the frequency of backslips. Further, analysis of two pathogenic mutations suggests that the reduced microtubule binding of KIF1C does not impair unloaded speed, in contrast to KIF1A mutations. However, force generation is severely impaired in these mutations, suggesting that KIF1C binds microtubules more strongly than KIF1A to generate higher forces and be less prone to backward slipping. When microtubule interactions are weakened, the motor can still move fast, as does KIF1A, but will not be able to generate substantial forces against a backward load. Our work sets a baseline to correlate changes in the biophysical properties of disease mutations in KIF1C with specific defects in neuronal cargo delivery and organelle distribution, defects in neuronal function and survival, time of onset, and the severity of the disease presentation in patients. This would lead to a detailed understanding of the disease etiology and offer a starting point to develop treatment strategies that delay or, indeed, reverse the symptoms.

STAR★METHODS

Detailed methods are provided in the online version of this paper and include the following:

- KEY RESOURCES TABLE
- RESOURCE AVAILABILITY
  - Lead contact
  - Materials availability
  - Data and code availability
- EXPERIMENTAL MODEL AND SUBJECT DETAILS
- METHOD DETAILS
  - Protein expression and purification
  - Force measurements

Figure 4. Pathogenic mutations of KIF1C impair intracellular cargo transport

(A) RPE1 cells treated with KIF1C siRNA and expressing either RNAi-protected wild-type KIF1C-GFP, $KIF1C_{P176L}$-GFP, or $KIF1C_{R169W}$-GFP. Motors accumulate in the tip of cell tails.

(B) Superplot of four cell tail accumulation experiments showing ratio of intensity in cell tails versus perinuclear cytoplasm. Small circles represent one cell and large circles the average of the experiment. Box plots show quartiles and 10/90th percentile whiskers of pooled data. n = 172, 133, and 186 cells, respectively. ***$p < 0.0001$ (Kruskal-Wallis test, Conover's test post hoc with Holm correction).

(C) Cellular transport assay based on rapamycin-induced heterodimerization of FKBP and FRB. Upon drug addition, FRB and mCherry-tagged KIF1C motors are recruited to mitochondria labeled with FKBP and GFP through fusion with the outer mitochondrial membrane protein monoamine oxidase (MAO).

(D) Mitochondrial morphology changes upon KIF1C recruitment in RPE1 cell expressing KIF1C-mCherry-FRB and FKBP-GFP-MAO. GFP channel shown, indicated times relative to rapamycin addition. Orange box indicates location of zoomed region. Orange arrows indicate membrane tubules pulled out, and blue arrowheads indicate beads formed.

(E) RPE1 cell co-expressing FKBP-GFP-MAO and either wild-type KIF1C-mCherry-FRB, $KIF1C_{P176L}$-mCherry-FRB, or $KIF1C_{R169W}$-mCherry-FRB. Left panel shows KIF1C signal, other panels MAO-labeled mitochondria. Time from rapamycin addition indicated in minutes. Scale bars, 20 μm.

(F) Averaged linescans showing mitochondrial redistribution upon recruitment of wild-type KIF1C, P176L, or R169W mutant. Cell position normalized to 0 in cell center and 1 at cell periphery. Errors show SEM. n = 78–110 cells, as indicated. Time color-coded as indicated on top of panel.

(G) Ratio of GFP-MAO intensity at the cell periphery versus perinuclear area over time. Errors show SEM. n as indicated in (F).

- ○ Single molecule motility assays
- ○ KIF1C localisation and mitochondria transport assays
- ● QUANTIFICATION AND STATISTICAL ANALYSIS

SUPPLEMENTAL INFORMATION

Supplemental information can be found online at https://doi.org/10.1016/j.cub.2022.07.029.

ACKNOWLEDGMENTS

The authors thank Nicholas Carter and CAMDU (Computing and Advanced Microscopy Unit) for access to the optical trap and microscopy support, Alice Bachmann for generating the $KIF1C_{R169W}$-GFP construct, Lewis Mosby for help with curve fitting, and Vishakha Karnawat for obtaining unloaded KHC motility data.

This work was funded by a research prize from the Lister Institute of Preventive Medicine and a Wellcome Trust Investigator Award (200870/Z/16/Z) to A.S., a Lister Institute Summer Studentship and a Chancellor's International PhD Scholarship from the University of Warwick that supported N.S., and a Ph.D. studentship from the MRC Doctoral Training Partnership (MR/N014294/1) that supported A.J.Z. D.R. was supported in part by the University of Warwick. A.T. was supported by a Wellcome Senior Investigator Award (103895/Z/14/Z) to R.A.C.

AUTHOR CONTRIBUTIONS

A.S. and N.S. conceived the project. N.S. cloned and purified KIF1C WT and mutants. D.R. cloned mammalian expression constructs, performed single-molecule experiments, and analyzed data. A.T. purified KHC. A.T. and N.S. performed optical trapping experiments and analyzed the data. A.S. performed cellular localization and transport assays. A.J.Z. analyzed single-molecule motility and cellular transport data. A.S. wrote the manuscript, with contributions from all authors.

DECLARATION OF INTERESTS

The authors declare no competing interests.

INCLUSION AND DIVERSITY

One or more of the authors of this paper self-identifies as an underrepresented ethnic minority in science. One or more of the authors of this paper self-identifies as a member of the LGBTQ+ community. One or more of the authors of this paper self-identifies as living with a disability.

Received: April 1, 2022
Revised: June 24, 2022
Accepted: July 13, 2022
Published: August 11, 2022

REFERENCES

1. Lipka, J., Kapitein, L.C., Jaworski, J., and Hoogenraad, C.C. (2016). Microtubule-binding protein doublecortin-like kinase 1 (DCLK1) guides kinesin-3-mediated cargo transport to dendrites. EMBO J. *35*, 302–318. https://doi.org/10.15252/embj.201592929.
2. Schlager, M.A., Kapitein, L.C., Grigoriev, I., Burzynski, G.M., Wulf, P.S., Keijzer, N., de Graaff, E., Fukuda, M., Shepherd, I.T., Akhmanova, A., and Hoogenraad, C.C. (2010). Pericentrosomal targeting of Rab6 secretory vesicles by bicaudal-D-related protein 1 (BICDR-1) regulates neuritogenesis. EMBO J. *29*, 1637–1651. https://doi.org/10.1038/emboj.2010.51.
3. Lee, P.L., Ohlson, M.B., and Pfeffer, S.R. (2015). Rab6 regulation of the kinesin family KIF1C motor domain contributes to Golgi tethering. eLife *4*, e06029. https://doi.org/10.7554/eLife.06029.
4. Theisen, U., Straube, E., and Straube, A. (2012). Directional persistence of migrating cells requires Kif1C-mediated stabilization of trailing adhesions. Dev. Cell *23*, 1153–1166. https://doi.org/10.1016/j.devcel.2012.11.005.
5. Marchionni, E., Méneret, A., Keren, B., Melki, J., Denier, C., Durr, A., Apartis, E., Boespflug-Tanguy, O., and Mochel, F. (2019). KIF1C variants are associated with hypomyelination, ataxia, tremor, and dystonia in fraternal twins. Tremor Other Hyperkinet. Mov. *9*, https://doi.org/10.7916/tohm.v0.641.
6. Yücel-Yılmaz, D., Yücesan, E., Yalnızoğlu, D., Oğuz, K.K., Sağıroğlu, M.Ş., Özbek, U., Serdaroğlu, E., Bilgiç, B., Erdem, S., İşeri, S.A.U., et al. (2018). Clinical phenotype of hereditary spastic paraplegia due to KIF1C gene mutations across life span. Brain Dev. *40*, 458–464. https://doi.org/10.1016/j.braindev.2018.02.013.
7. Caballero Oteyza, A., Battaloğlu, E., Ocek, L., Lindig, T., Reichbauer, J., Rebelo, A.P., Gonzalez, M.A., Zorlu, Y., Ozes, B., Timmann, D., et al. (2014). Motor protein mutations cause a new form of hereditary spastic paraplegia. Neurology *82*, 2007–2016. https://doi.org/10.1212/WNL.0000000000000479.
8. Dor, T., Cinnamon, Y., Raymond, L., Shaag, A., Bouslam, N., Bouhouche, A., Gaussen, M., Meyer, V., Durr, A., Brice, A., et al. (2014). KIF1C mutations in two families with hereditary spastic paraparesis and cerebellar dysfunction. J. Med. Genet. *51*, 137–142. https://doi.org/10.1136/jmedgenet-2013-102012.
9. Dorner, C., Ullrich, A., Häring, H.-U., and Lammers, R. (1999). The kinesin-like motor protein KIF1C occurs in intact cells as a dimer and associates with proteins of the 14–3-3 family. J. Biol. Chem. *274*, 33654–33660. https://doi.org/10.1074/jbc.274.47.33654.
10. Siddiqui, N., Zwetsloot, A.J., Bachmann, A., Roth, D., Hussain, H., Brandt, J., Kaverina, I., and Straube, A. (2019). PTPN21 and Hook3 relieve KIF1C autoinhibition and activate intracellular transport. Nat. Commun. *10*, 2693. https://doi.org/10.1038/s41467-019-10644-9.
11. Block, S.M., Goldstein, L.S., and Schnapp, B.J. (1990). Bead movement by single kinesin molecules studied with optical tweezers. Nature *348*, 348–352.
12. Carter, N.J., and Cross, R.A. (2005). Mechanics of the kinesin step. Nature *435*, 308–312. https://doi.org/10.1038/nature03528.
13. Coy, D.L., Wagenbach, M., and Howard, J. (1999). Kinesin takes one 8-nm step for each ATP that it hydrolyzes. J. Biol. Chem. *274*, 3667–3671.
14. Toleikis, A., Carter, N.J., and Cross, R.A. (2020). Backstepping mechanism of Kinesin-1. Biophys. J. *119*, 1984–1994. https://doi.org/10.1016/j.bpj.2020.09.034.
15. Kojima, H., Muto, E., Higuchi, H., and Yanagida, T. (1997). Mechanics of single kinesin molecules measured by optical trapping nanometry. Biophys. J. *73*, 2012–2022. https://doi.org/10.1016/S0006-3495(97)78231-6.
16. Khalil, A.S., Appleyard, D.C., Labno, A.K., Georges, A., Karplus, M., Belcher, A.M., Hwang, W., and Lang, M.J. (2008). Kinesin's cover-neck bundle folds forward to generate force. Proc. Natl. Acad. Sci. USA *105*, 19247–19252.
17. Budaitis, B.G., Jariwala, S., Rao, L., Yue, Y., Sept, D., Verhey, K.J., and Gennerich, A. (2021). Pathogenic mutations in the kinesin-3 motor KIF1A diminish force generation and movement through allosteric mechanisms. J. Cell Biol. *220*. e202004227.
18. Svoboda, K., Schmidt, C.F., Schnapp, B.J., and Block, S.M. (1993). Direct observation of kinesin stepping by optical trapping interferometry. Nature *365*, 721–727. https://doi.org/10.1038/365721a0.
19. McHugh, T., Drechsler, H., McAinsh, A.D., Carter, N.J., Cross, R.A., and Pollard, T.D. (2018). Kif15 functions as an active mechanical ratchet. Mol. Biol. Cell *29*, 1743–1752. https://doi.org/10.1091/mbc.E18-03-0151.
20. Okada, Y., Higuchi, H., and Hirokawa, N. (2003). Processivity of the single-headed kinesin KIF1A through biased binding to tubulin. Nature *424*, 574–577. https://doi.org/10.1038/nature01804.

21. Reinemann, D.N., Norris, S.R., Ohi, R., and Lang, M.J. (2018). Processive Kinesin-14 HSET exhibits directional flexibility depending on motor traffic. Curr. Biol. *28*, 2356–2362.e5. https://doi.org/10.1016/j.cub.2018.06.055.

22. Korneev, M.J., Lakämper, S., and Schmidt, C.F. (2007). Load-dependent release limits the processive stepping of the tetrameric Eg5 motor. Eur. Biophys. J. *36*, 675–681. https://doi.org/10.1007/s00249-007-0134-6.

23. Lam, A.J., Rao, L., Anazawa, Y., Okada, K., Chiba, K., Dacy, M., Niwa, S., Gennerich, A., Nowakowski, D.W., and McKenney, R.J. (2021). A highly conserved 310 helix within the kinesin motor domain is critical for kinesin function and human health. Sci. Adv. *7*, eabf1002.

24. Sudhakar, S., Abdosamadi, M.K., Jachowski, T.J., Bugiel, M., Jannasch, A., and Schäffer, E. (2021). Germanium nanospheres for ultraresolution picotensiometry of kinesin motors. Science *371*, https://doi.org/10.1126/science.abd9944.

25. Soppina, V., Norris, S.R., Dizaji, A.S., Kortus, M., Veatch, S., Peckham, M., and Verhey, K.J. (2014). Dimerization of mammalian kinesin-3 motors results in superprocessive motion. Proc. Natl. Acad. Sci. USA *111*, 5562–5567. https://doi.org/10.1073/pnas.1400759111.

26. Scarabelli, G., Soppina, V., Yao, X.-Q., Atherton, J., Moores, C.A., Verhey, K.J., and Grant, B.J. (2015). Mapping the processivity determinants of the Kinesin-3 motor domain. Biophys. J. *109*, 1537–1540. https://doi.org/10.1016/j.bpj.2015.08.027.

27. Zaniewski, T.M., Gicking, A.M., Fricks, J., and Hancock, W.O. (2020). A kinetic dissection of the fast and superprocessive kinesin-3 KIF1A reveals a predominant one-head-bound state during its chemomechanical cycle. J. Biol. Chem. *295*, 17889–17903. https://doi.org/10.1074/jbc.RA120.014961.

28. Rogers, K.R., Weiss, S., Crevel, I., Brophy, P.J., Geeves, M., and Cross, R. (2001). KIF1D is a fast non-processive kinesin that demonstrates novel K-loop-dependent mechanochemistry. EMBO J *20*, 5101–5113. https://doi.org/10.1093/emboj/20.18.5101.

29. Atherton, J., Farabella, I., Yu, I.M., Rosenfeld, S.S., Houdusse, A., Topf, M., and Moores, C.A. (2014). Conserved mechanisms of microtubule-stimulated ADP release, ATP binding, and force generation in transport kinesins. eLife *3*, e03680. https://doi.org/10.7554/eLife.03680.

30. Boyle, L., Rao, L., Kaur, S., Fan, X., Mebane, C., Hamm, L., Thornton, A., Ahrendsen, J.T., Anderson, M.P., Christodoulou, J., et al. (2021). Genotype and defects in microtubule-based motility correlate with clinical severity in KIF1A-associated neurological disorder. HGG Adv *2*, 100026.

31. Woehlke, G., Ruby, A.K., Hart, C.L., Ly, B., Hom-Booher, N., and Vale, R.D. (1997). Microtubule interaction site of the kinesin motor. Cell *90*, 207–216. https://doi.org/10.1016/s0092-8674(00)80329-3.

32. Efimova, N., Grimaldi, A., Bachmann, A., Frye, K., Zhu, X., Feoktistov, A., Straube, A., and Kaverina, I. (2014). Podosome-regulating kinesin KIF1C translocates to the cell periphery in a CLASP-dependent manner. J. Cell Sci. *127*, 5179–5188. https://doi.org/10.1242/jcs.149633.

33. Wong, M., and Munro, S. (2014). Membrane trafficking. The specificity of vesicle traffic to the Golgi is encoded in the golgin coiled-coil proteins. Science *346*, 1256898.

34. Schindelin, J., Arganda-Carreras, I., Frise, E., Kaynig, V., Longair, M., Pietzsch, T., Preibisch, S., Rueden, C., Saalfeld, S., Schmid, B., et al. (2012). Fiji: an open-source platform for biological-image analysis. Nat. Methods *9*, 676–682. https://doi.org/10.1038/nmeth.2019.

35. Trowitzsch, S., Bieniossek, C., Nie, Y., Garzoni, F., and Berger, I. (2010). New baculovirus expression tools for recombinant protein complex production. J. Struct. Biol. *172*, 45–54.

CellPress
OPEN ACCESS

STAR★METHODS

KEY RESOURCES TABLE

REAGENT or RESOURCE	SOURCE	IDENTIFIER
**Chemicals, peptides, and recombinant proteins**		
X-rhodamine-labelled tubulin	Cytoskeleton Inc.	TL6290M
HiLyte Fluor 647-labelled tubulin	Cytoskeleton Inc.	TL670M
Biotin-tubulin	Cytoskeleton Inc.	T333P
PLL(20)-g[3.5]-PEG(2)/PEG(3.4)-Biotin (50%)	Susos AG	PLL(20)-g[3.5]-PEG(2)/PEGbi
streptavidin	Sigma-Aldrich / Merck	S4762
κ-casein	Sigma-Aldrich / Merck	C0406
phosphocreatine	Sigma-Aldrich / Merck	P7936
creatine phosphokinase	Sigma-Aldrich / Merck	C3755
catalase	Sigma-Aldrich / Merck	C9322
glucose oxidase	Sigma-Aldrich / Merck	G7141
N,N,N′,N′-Tetramethylethylenediamine	Sigma-Aldrich / Merck	T9281
Acrylamide/Bis-acrylamide, 30% solution	Sigma-Aldrich / Merck	A3699
Ex-Cell 420 serum-free media	Sigma-Aldrich / Merck	14420C
Penicillin-Streptomycin	Sigma-Aldrich / Merck	P0781
L-glutamine	Sigma-Aldrich / Merck	L7513
Dulbecco's Modified Eagle's Medium/Nutrient Mixture F-12 Ham	Sigma-Aldrich / Merck	D6421
Sodium bicarbonate solution	Sigma-Aldrich / Merck	S8761
Trypsin-EDTA solution	Sigma-Aldrich / Merck	T3924
Glycerol	Thermofisher	15514-011
Kanamycin	Sigma-Aldrich / Merck	K4000
Tetracycline	Sigma-Aldrich / Merck	T3258
Gentamicin	Sigma-Aldrich / Merck	G1272
IPTG	Melford	MB1008
X-Gal	Melford	MB1001
Escort-IV	Sigma-Aldrich / Merck	L-3287
Imidazole	Sigma-Aldrich / Merck	I2399
Ni NTA Agarose	Qiagen	30230
DTT	Sigma-Aldrich / Merck	D9779
Pipes	Sigma-Aldrich / Merck	RES0703P-A103X
ATP	Roche	10127531001
**Experimental models: Cell lines**		
Spodoptera frugiperda Sf9 cells	VWR	EM71104-3
hTERT RPE1 (human retinal pigment epithelia)	ATCC	CRL-4000
**Oligonucleotides**		
pFB5' (5'-GATTACGATATCCCAACGACC-3')	This study	N/A
AS358 (5'-GAAGGGATCCACAGTTCCCCCATCCTC-3')	This study	N/A
AS359 (5'- GGGGATCCCCTCGTTCCCGTTCC-3')	This study	N/A
AS376 (5'-CTGCGGGTC<u>T</u>GGGAGCACC-3')	This study	N/A
AS528 (5'-CTGCACGTAC<u>A</u>GGCCCAGGATG-3')	This study	N/A
UT01 (5'-GGAATTCTGGAGCTATGGCTGGTG-3')	This study	N/A
UT170 (5'-ACTGACCTTCTCCGAGTCC-3')	This study	N/A
siRNA targeting sequence KIF1C #2 GUGAGCUAUAU GGAGAUCU-[dA]-[dC]	Theisen et al.[4]	N/A

(*Continued on next page*)

Continued		
REAGENT or RESOURCE	SOURCE	IDENTIFIER
Recombinant DNA		
pFastBac-M13-6His-KIF1C-GFP	Siddiqui et al.[10]/Addgene	RRID:Addgene_130975
pFastBac-M13-6His-KIF1C$_{P176L}$-GFP	This study/Addgene	RRID:Addgene_185978
pFastBac-M13-6His-KIF1C$_{R169W}$-GFP	This study / Addgene	RRID:Addgene_185979
pKIF1C$_{RIP2}$-GFP	Efimova et al.[32]/Addgene	RRID:Addgene_130977
pKIF1C$_{P176L}$-GFP	This study/Addgene	RRID:Addgene_185980
pKIF1C$_{R169W}$-GFP	This study / Addgene	RRID:Addgene_185981
pβactin-KIF1C_MD-GFP-FRB	Lipka et al.[1]	N/A
pKIF1C-mCherry-FRB	This study / Addgene	RRID:Addgene_185982
pKIF1C$_{P176L}$-mCherry-FRB	This study / Addgene	RRID:Addgene_185983
pKIF1C$_{R169W}$-mCherry-FRB	This study / Addgene	RRID:Addgene_185984
GFP-Myc-MAO	Wong and Munro[33]	N/A
Software and algorithms		
Fiji / ImageJ	Schindelin et al.[34]	https://imagej.net/software/fiji/
R	The R foundation	https://www.R-project.org
Matlab	Mathworks	https://uk.mathworks.com/products/matlab.html
Python	The Python Software Foundation	https://www.python.org/
GaussFit_OnSpot	Peter Haub & Tobias Meckel, ImageJ plugin	https://imagej.nih.gov/ij/plugins/gauss-fit-spot/index.html
plotSpread	Jonas Dorn, MATLAB Central file exchange	https://www.mathworks.com/matlabcentral/fileexchange/37105-plot-spread-points-beeswarm-plot
Other		
Optical Trap	Carter and Cross[12]	N/A
Multiline TIRF microscope	Olympus	N/A
Deltavision widefield deconvolution microscope	Applied Precision / Imsol	https://warwick.ac.uk/fac/sci/med/research/biomedical/facilities/camdu/microscopes/dv1/dv1methodsreporting/
AKTA purifier	Cytvia	https://www.marshallscientific.com/GE-AKTA-Purifier-10-FPLC-System-p/ak-p10.htm

RESOURCE AVAILABILITY

Lead contact

Further information and requests for resources and reagents should be directed to and will be fulfilled by the lead contact, Anne Straube (anne@mechanochemistry.org).

Materials availability

All plasmids generated in this study have been deposited to Addgene under ID numbers 185978–185984.

Data and code availability

- All data reported in this paper will be shared by the lead contact upon request.
- All original code is available in this paper's supplemental information.
- Any additional information required to reanalyse the data reported in this paper is available from the lead contact upon request.

EXPERIMENTAL MODEL AND SUBJECT DETAILS

Recombinant protein expression was performed in Sf9 insect cells (VWR). Sf9 cells were maintained in Ex-Cell 420 serum-free media (Sigma, 14420C) at a pre-log density of 0.5-0.75 × 10^6 cells/ml at 29 °C.

Cell biology experiments were performed in hTERT RPE1 (ATCC) cells. hTERT RPE1 cells were grown in DMEM/Nutrient F-12 Ham (Sigma, D6421) supplemented with 10% FBS (Sigma), 2 mM L-glutamine (Sigma), 100 U/ml penicillin (Sigma), 100 μg/ml streptomycin (Sigma) and 2.3 g/l sodium bicarbonate (#S8761 Sigma) in a humidified incubator at 37°C and 8% CO_2.

METHOD DETAILS

Protein expression and purification

pFastBac-M13-6His-KIF1C-GFP was described previously[10] and is available from Addgene (130975). The human patient mutations P176L[7] and R169W[8] were introduced into this plasmid as follows. *pFastBac-M13-KIF1CGFP*$_{P176L}$ was generated using *pFastBac-M13-6His-KIF1C-GFP* as template in a three-step mutagenesis PCR with upstream primer pFB5′ (5′-GATTACGATATCCCAAC GACC-3’), downstream primer AS358 (5’-GAAGGGATCCACAGTTCCCCCATCCTC-3’) and mutagenesis primer AS528 (5’-CTGCACGTAC**A**GGCCCAGGATG-3’). The fragment containing the mutation was replaced in *pFastBac-M13-6His-KIF1C-GFP* using *Asc*I and *Stu*I. *pFastBac-M13-6His-KIF1C-GFP*$_{R169W}$ was generated similarly using upstream primer pFB5’, downstream primer UT170 (5’-ACTGACCTTCTCCGAGTCC-3’) and mutagenesis primer AS376 (5’-CTGCGGGTCT**T**GGGAGCACC-3’). The fragment containing the mutation was replaced in *pFastBac-M13-6His-KIF1C-GFP* using *Asc*I and *Bsi*WI.

Purification of full length human KIF1C in insect cells was performed as described previously[10] Briefly, pFastBac-M13-6His-KIF1C-GFP, pFastBac-M13-6His-KIF1C$_{P176L}$-GFP, and pFastBac-M13-6His-KIF1C$_{R169W}$-GFP plasmids were transformed into DH10BacYFP competent cells[35] and plated on LB-Agar supplemented with 30 μg/ml kanamycin (#K4000, Sigma), 7 μg/ml gentamycin (#G1272, Sigma), 10 μg/ml tetracycline (#T3258, Sigma, 40 μg/ml Isopropyl β-D-1- thiogalactopyranoside (IPTG, #MB1008, Melford) and 100 μg/ml X-Gal (#MB1001, Melford). Positive transformants (white colonies) were screened by PCR using M13 forward and reverse primers for the integration into the viral genome. The bacmid DNA was isolated from the positive transformants by the alkaline lysis method and transfected into SF9 cells (VWR, #EM71104-3) with Escort IV (#L-3287, Sigma) according to the manufacturer’s protocol. After 5–7 days, the virus (passage 1, P1) was harvested by centrifugation at 300 × g for 5 min in a swing out 5804 S-4-72 rotor (Eppendorf). Baculovirus infected insect cell (BIIC) stocks were made by infecting SF9 cells with P1 virus and freezing cells before lysis (typically around 36 h) in a 1° cooling rate rack (#NU200 Nalgene) at −80 °C. P1 virus was propagated to passage 2 (P2) by infecting 50 ml of SF9 (VWR, #EM71104-3) culture and harvesting after 5–7 days as described above. For large-scale expression, 500 ml of SF9 cells at a density of 1–1.5 × 10^6 cells/ml were infected with one vial of BIIC or P2 virus. Cells were harvested when 90% infection rate was achieved as observed by YFP fluorescence, typically between 48 and 72 h. Cells were pelleted at 252 × g in a SLA-3000 rotor (Thermo Scientific) for 20 min. The pellet was resuspended in 4 ml of SF9 lysis buffer (50 mM Sodium phosphate pH 7.5, 150 mM NaCl, 20 mM Imidazole, 0.1% Tween 20, 1.5 mM MgCl2) per gram of cell pellet, supplemented with 0.1 mM ATP and cOmplete protease inhibitor cocktail (#05056489001, Roche) and lysed using a douncer (#885301, Kontes) with 20 strokes. Lysates were then cleared by centrifugation at 38,000 × g in a SS-34 rotor (Sorvall) for 30 min, or 200,000 × g for 40 min in a T865 rotor (Sorvall). SP Sepharose beads (#17-0729-01, GE Healthcare) were equilibrated with the lysis buffer and the cleared lysate obtained is mixed with the beads and batch bound for 1 h. Next, the beads were loaded onto a 5 ml disposable polypropylene gravity column (#29922, Thermo Scientific) and washed with at least 10 CV SP wash buffer (50 mM sodium phosphate pH 7.5, 150 mM NaCl) and eluted with SP elution buffer (50 mM sodium phosphate pH 7.5, 300 mM NaCl). The peak fractions obtained were pooled and diluted with Ni-NTA lysis buffer (50 mM sodium phosphate pH 7.5, 150 mM NaCl, 20 mM Imidazole, 10% glycerol) and batch bound to Ni-NTA beads (#30230, Qiagen) for 1 h. The beads were loaded onto a gravity column and washed with at least 10 CV of Ni-NTA wash buffer (50 mM sodium phosphate pH 7.5, 150 mM NaCl, 50 mM imidazole and 10% glycerol) and eluted with Ni-NTA elution buffer (50 mM sodium phosphate pH 7.5, 150 mM NaCl, 150 mM Imidazole, 0.1 mM ATP and 10% glycerol). The peak fractions were run on an SDS-PAGE gel for visualisation and protein was aliquoted, flash frozen and stored in liquid nitrogen.

Recombinant Kinesin Heavy Chain from *Drosophila melanogaster* was purified as described previously.[13] Briefly, BL21 DE3 (Invitrogen) were transformed with plasmid pPK113-6H-DHK (accession # AF053733), and the cells were grown at 37 °C until OD_{600nm} reached 0.5. Expression was induced with 0.4 mM Isopropyl β-D-1-thiogalactopyranoside at 15 °C overnight. Cells were harvested by centrifugation (3000 × *g*, 15 min, RT). Cell pellets were stored at -80 °C. For purification, pellets were thawed on ice, and cells were lysed by sonication in buffer A (50 mM phosphate buffer pH 7.5, 300 mM NaCl, 10% glycerol, 1 mM $MgCl_2$, 0.1 mM ATP, and 40 mM Imidazole). The lysate was clarified by centrifugation (100,000 × *g*, T865 rotor, 30 min, 4 °C) and the supernatant was then loaded onto 1 ml HisTrap HP column (Qiagen) at 4 °C. Unbound protein was washed with buffer A containing 90 mM imidazole. Finally, the protein was eluted over 300 mM imidazole gradient (10 column volumes). The peak fractions were run on an SDS-PAGE gel for visualisation and protein was aliquoted, flash frozen and stored in liquid nitrogen.

Force measurements

560 nm Polystyrene beads (Polysciences) and motor protein were incubated together in 80 mM PIPES pH 7, 2 mM $MgSO_4$, 1 mM EGTA, 1 mM DTT, 3 mg/ml D-Glucose, 0.2 mg/ml casein and 1 mM ATP. The concentration of motor was decreased such that only 20-30% of the beads moved. Flow cell coverslips were plasma-cleaned and functionalized with APTES silane. Two coverslips were stuck together with Dow Corning High Vacuum Grease. Two lines of grease were applied to the base coverslip (22 × 50 mm) using a syringe, the top coverslip (22 × 22 mm) was placed, forming a flow cell of approximately 10 μl capacity. For covalent attachment of microtubules, glutaraldehyde (8%) was added to the flow cell and incubated for 30 min. The flow cell was washed with MilliQ water. The microtubules were diluted to the required concentration and introduced to the flow cell and allowed to adsorb onto the surface. The microtubules were incubated for an hour to allow for covalent attachment. Next, 0.2 μl of bead-motor protein solution was diluted in 20 μl of assay buffer composed of BRB80, 1 mM ATP, 0.4 mg/ml casein, 10 μM taxol and 0.4 μl oxygen scavenger mix (1 mg/ml catalase, 5 mg/ml Glucose oxidase, 50% glycerol). The beads were then flown to the cell and microtubules were visualised using in-built differential interference contrast on the Optical Trap setup[12] at room temperature of 22°C. The trap was steered to

position the bead above the microtubule and the image was projected onto the quadrant photodiode detector. Bead positions were acquired at 20 kHz for 180 s, trap stiffness 0.065 to 0.075 pN nm^{-1} for KHC, 0.06 to 0.067 pN nm^{-1} for wildtype KIF1C-GFP, 0.01 to 0.052 pN nm^{-1} for $KIF1C_{P176L}$-GFP and 0.014 pN nm^{-1} for $KIF1C_{R169W}$-GFP. A calibration for trap stiffness was done each day before commencement of measurements. Measurements were done under conditions when 20% or less beads were running on microtubules. According to a Poisson distribution, about 12% of moving beads could have more than one motor capable of engaging with the microtubule. The rare traces that showed unusual high force events that suggested more than one motor is engaged were excluded from the analysis. For wildtype KIF1C-GFP, data was pooled from 38 recordings of 7 motile beads from 3 independent experiments. For $KIF1C_{P176L}$-GFP, data pooled from 21 recordings of 6 motile beads from 4 independent experiments. For $KIF1C_{R169W}$-GFP data pooled from 3 recordings of 2 motile beads from 1 experiment. KHC data are a subset of a previously published dataset.[14] The analysis only included data that were acquired at a comparable trap stiffness to KIF1C data (8 recordings from 8 different beads, 8 microtubules acquired on three different experiment days). In addition, data from superstall conditions were included for the analysis of fore/backstep ratios.

Data analysis was carried out on baseline-corrected traces. From these traces, the number of force events per MT encounter was determined by counting the number of times the motor moved forwards and slipped back for at least 24 nm before detachment. Events where the bead returned to the trap centre but restarted immediately were considered as long backslips rather than detachments and thus considered as the same MT encounter in this analysis.

An automatic run-event analysis using custom code written in R (see Methods S1) was used to determine the peak force, duration of force-generation events, the force-velocity curve and the restart times between events. Data was smoothed by 2000-point averaging (100 ms) and events above 0.5 pN force, lasting more than 200 ms, were included. The end of the run was registered when the event returned below the 0.5 pN threshold. Force-velocity relationship was determined from linear fits to the displacement versus time data smoothed by 200 points (10 ms) over 0.5 pN force bins. Unloaded velocities were determined from DIC videos of beads moving along microtubules after the trapping laser was switched off. DIC videos were converted to digital timelapse movies at 5 frames per second and speeds were obtained from kymographs using a custom macro in ImageJ (see Methods S1).

Stall force was determined manually from each identified force event as the average force that was maintained with fluctuations of no more than one (8 nm) step before the bead returned to the trap center or slipped backwards for at least 24 nm. Only stalls of at least 10 ms duration were included in the analysis.

For the step analysis, a moving window t-test algorithm[12] was used with the following parameters: t-test score threshold=30, minimum step size=5 nm, minimum force=1 pN, moving average, n=20 (1 ms) (see Methods S1). The size of the window and the threshold were varied to determine accurate steps. When the bead returned to within 1.2 pN of the centre of the trap, we designated this as a detachment. Other movements towards the trap centre were designated as backslips. To determine the stall force defined as the force at which the motor undertakes as many forward steps as backslips, we only considered backslips of up to 12 nm for the fore/back ratio as their probability of detection is comparable to forward steps.

Single molecule motility assays

Microtubules were assembled from 5 μl of 18 mg/ml unlabelled pig tubulin, 0.2 μl of 1 mg/ml HiLyte Fluor 647 or X-rhodamine-labelled tubulin (#TL670M, #TL6290M, Cytoskeleton) and 0.5 μl of 0.5 mg/ml biotin tubulin (#T333P, Cytoskeleton) in 15 μl MRB80 (80 mM PIPES pH 6.8, 4 mM MgCl2, 1 mM EGTA, 1 mM DTT) supplemented with 4 mM GTP. The mixture was incubated at 37°C for 90 min before diluting in 80 μl MRB80 supplemented with 20 μM Taxol. Unincorporated tubulin was removed by pelleting microtubules through a glycerol cushion (30% glycerol in MRB80) at 20,238 × g for 12 min at room temperature. The microtubule pellet was resuspended in 80 μl of MRB80 with 20 μM Taxol and stored at 28°C covered from light for use on the same day.

Coverslips (22 × 22) were cleaned by incubating in 2.3 M hydrochloric acid overnight at 60 °C. The next day, coverslips were washed with Millipore water and sonicated at 60 °C for 5 min. The wash cycle was repeated five times. The coverslips were dried using a Spin Clean (Technical video) and plasma cleaned using Henniker plasma clean (HPT-200) for 3 min. Flow chambers were made using clean glass slides (Menzel Gläser Superfrost Plus, Thermo Scientific) and double- sided sticky tape (Scotch 3 M) by placing the cleaned coverslip on the sticky tape creating a 100 μm deep flow chamber. The surface was coated with (0.2 mg/ml) PLL(20)-g[3.5]-PEG(2)/PEG(3.4)-Biotin (50%) (#PLL(20)-g[3.5]-PEG(2)/PEGbi, Susos AG). Biotin-647-microtubules were attached to this surface with streptavidin (0.625 mg/ml) (#S4762 Sigma) and the surface was blocked with κ-casein (1 mg/ml) (#C0406 Sigma).

KIF1C-GFP, $KIF1C_{P176L}$-GFP and $KIF1C_{R169W}$-GFP were diluted in TIRF Assay Buffer (25 mM HEPES-KOH pH 7.2, 5 mM MgSO4, 1 mM EGTA, 1 mM DTT, 10 μM Taxol) supplemented with 0.05% Tween-20, 25 mM KCl and 200ng/ml κ-casein, spun at 100,000 × g for 10 min in an Airfuge (Beckman Coulter) and fluorescence at 507 nm measured using a NanoDrop™ 3300 Fluorospectrometer to verify concentrations used in the assay and determine landing rates. Proteins were then added to the motility mix (TIRF Assay Buffer supplemented with 5 mM ATP, 5 mM phosphocreatine (#P7936, Sigma), 7 U/ml creatine phosphokinase (#C3755 Sigma), 0.2 mg/ml catalase, 0.4 mg/ml glucose oxidase, 4 mM DTT, 50 mM glucose (#G8270, Sigma), 25 mM KCl, 10 μM taxol, 0.2 mg/ml κ-casein) and flown into the chamber.

Chambers were observed on an Olympus TIRF system using a ×100 NA 1.49 objective, 488 and 640 nm laser lines, an ImageEM emCCD camera (Hamamatsu Photonics) under the control of xCellence software (Olympus), an environmental chamber maintained at 25 °C (Okolab, Ottaviano, Italy). Images were acquired at 5 fps for 180 seconds using 2x2 binning, thus the resulting images have 162 nm pixels and 200 ms temporal resolution.

The fraction of microtubules that contain one or more kinesin motors at the plus end was determined by counting all microtubules for which both ends were visible in the first frame of the second movie taken from each chamber, i.e. about 3 minutes after start of the experiment, and scoring for the presence / absence of a GFP signal at one of the ends.

Motility was analysed by tracing microtubules and generating maximum intensity kymographs from 7 pixel wide lines using the ImageJ Kymograph plugin from Arne Seitz (EPFL Lausanne). Motor tracks from kymographs were traced by hand and recorded as ImageJ ROIs. These paths were analysed in a custom-built python analysis script. Individual phases of tracks were segmented from the ROI, and motors were said to be translocating towards the plus-end or minus-end of the microtubule in each phase if their speed towards that end of the microtubule was greater than 25 nm/s. When the motor's speed was less than 25 nm/s, it was annotated as paused. Motors with an average speed of less than 25 nm/s or a total run length of less than 500 nm were considered as static. Non-static motors that underwent bidirectional motion of at least 324 nm or just moved towards the minus end where classed as diffusing. Only the remaining, plus end directed motors were analysed for run parameters. Dwell times were calculated as the total time the motor spent on the microtubule until it reaches the plus end or the end of the recording. Run length is the total distance covered by the motor. The pause-corrected average speed was calculated by dividing the run length by the dwell time minus any time when the motor was paused. Superplots show the mean speed, run-length, or dwell time for a given experimental day, with smaller colour-coded spots showing the individual measured values of motors. On each of the three experiment days, one to four chambers were prepared for each protein. Statistics were calculated at the per-motor level between experimental groups. The data were tested for normality using D'Agostino and Pearson's test, and as they were not normally distributed, a Kruskal-Wallis test was used to determine if experimental groups differed significantly. Where a statistically significant difference was indicated, pairwise interactions were tested using Conover's post-hoc test and p-values were corrected for multiple comparisons using the Holm-Bonferroni method.

To test homogeneity of the motors in the motility assay, the intensity of motors was determined using the ImageJ plugin GaussFit_OnSpot for each line traced in the kymograph. The normalised intensity value output by the function for the first 10 time points of each track were averaged to obtain the fluorescent intensity for the motor. To pool intensity measurements from different experiments, data were divided by the average intensity of all wildtype KIF1C detections and then grouped into highly processive motors with a run length of more than 1 μm and motors that moved less than 1 μm. While a small fraction of motor clusters were observed in the assays, the clusters did not primarily contribute to the highly processive runs (Figure S2). Data were plotted in MATLAB using the plotSpread function from Jonas Dorn.

KIF1C localisation and mitochondria transport assays

pKIF1C$_{P176L}$-GFP and pKIF1C$_{R169W}$-GFP were based on pKIF1C$_{RIP2}$-GFP (available from Addgene 130977.[32] R169W was introduced using a mutagenesis PCR with upstream primer UT01 (5'-GGAATTCTGGAGCTATGGCTGGTG-3'), mutagenesis primer AS376 (5'-CTGCGGGTC**T**GGGAGCACC-3') and downstream primer UT170 (5'-ACTGACCTTCTCCGAGTCC-3'). The fragment containing the mutation was replaced in pKIF1C$_{RIP2}$-GFP using *Eco*RI and *Bsi*WI. P176L was introduced using a mutagenesis PCR with upstream primer UT01, mutagenesis primer AS528 (5'-CTGCACGTAC**A**GGCCCAGGATG-3') and downstream primer AS359 (5'- GGGGATCCCCTCGTTCCCGTTCC-3'). The fragment containing the mutation was replaced in pKIF1C$_{RIP2}$-GFP using *Eco*RI and *Bsp*EI. The RIP2 mutation contained in all these constructs are a series of silent mutations providing RNAi resistance for siRNA KIF1C #2 GUGAGCUAUAUGGAGAUCU-[dA]-[dC].

pKIF1C-mCherry-FRB, pKIF1C$_{P176L}$-mCherry-FRB and pKIF1C$_{R169W}$-mCherry-FRB plasmids were based on pβactin-KIF1C_MD-GFP-FRB, a kind gift from Caspar Hoogenraad.[1]The KIF1C motor domain was replaced with full length human KIF1C fused to mCherry from pKIF1C-mCherry (available from Addgene 130978[4]) using *Nhe*I and *Bsr*GI. The mutations were introduced from pKIF1C$_{P176L}$-GFP and pKIF1C$_{R169W}$-GFP using *Nhe*I and *Bam*HI. FKBP-GFP-Myc-MAO was a kind gift from Sean Munro (MRC-LMB, Cambridge).[33]

For tail localisation experiments, RPE1 cells seeded into fibronectin-coated glass-bottom dishes were transfected with validated KIF1C siRNA #2[4] using Oligofectamine (Invitrogen), transfected 24 hours later either with KIF1C-GFP, pKIF1C$_{P176L}$-GFP or pKIF1C$_{R169W}$-GFP using Fugene 6 (Promega) and imaged a further 24 hours later on an Olympus Deltavision microscope (Applied Precision, LLC) equipped with eGFP filter sets and a CoolSNAP HQ2 camera (Roper Scientific) under the control of SoftWorx (Applied Precision). 37°C and 5% CO_2 were maintained in a TOKAI Hit stage incubator. Images from migrating cells with a tail at the rear expressing moderate levels of the GFP constructs were acquired. To determine tail accumulation, average intensity measurements from tip of cell tails, the perinuclear area and background outside of the cell were taken. After background subtraction, the ratio of tail versus cytoplasm intensity was calculated. 4 experiments with a total of 133-186 cells were analysed for each condition. Statistical analysis was performed on the per cell level between experimental groups. A Kruskal-Wallis test was used to determine if experimental groups differed significantly. Where a statistically significant difference was indicated, pairwise interactions were tested using Conover's post-hoc test and p-values were corrected for multiple comparisons using the Holm-Bonferroni method.

For mitochondria transport experiments, RPE1 cells were co-transfected with 1 μg KIF1C-mCherry-FRB and 0.5 μg FKBP-GFP-Myc-MAO using Fugene 6 (Promega) in fibronectin-coated glass-bottom dishes and imaged 24 hours later on an Olympus Deltavision microscope (Applied Precision, LLC) equipped with eGFP, mCherry filter sets and a CoolSNAP HQ2 camera (Roper Scientific) under the control of SoftWorx (Applied Precision). Live cells were imaged using an Olympus 40x UPlanFL N Oil NA1.3 objective in imaging medium (Leibovitz's L-15 medium (Gibco™ 21083027) without phenol red, supplemented with 10% FBS (Sigma), 2 mM L-glutamine (Sigma), 100 U/ml penicillin (Sigma), 100 μg/ml streptomycin (Sigma)) at 37 °C. Cells expressing moderate levels of

both KIF1C and the mitochondrial marker were identified and their stage position labelled. Images in both channels were acquired from each stage position at 2-minute intervals for 22 minutes to determine organelle redistribution. After the first image from each cell, rapamycin was added to the imaging dish at a final concentration of 200 μM. To observe mitochondria morphology change and motility, images from a single cell were acquired at 2 s intervals for 10 minutes and rapamycin added after 30 seconds.

To quantify organelle redistribution, 33 pixel wide line profiles were acquired from the cell centre to the cell tips at each timepoint. The average intensity of the KIF1C signal along the entire line was used to include only cells with comparable expression level across the wildtype and mutant constructs. Positions along line profiles were normalised so that the cell centre was set to 0 and the cell tip to 1. This normalised distance was binned into 100 equal-width bins and intensity values of MAO for each cell were sorted into these bins. The centre of each bin was plotted against the average MAO intensity +/- the standard error of the binned values. To quantify mitochondria redistribution over time, periphery GFP-MAO signal (0.8 to 1.0) was divided by perinuclear GFP-MAO signal (0.0 to 0.2).

QUANTIFICATION AND STATISTICAL ANALYSIS

The number of experimental repeats, number of beads/cells/microtubules analysed is provided in the method details section. Information on the number of observations shown in each graph is provided in the figure legend. Error bars show either SD or SEM as indicated in the figure legend. Superplots show individual data points and mean values colour-coded for each experiment. Box plots show Median, quartiles and whiskers at 10th and 90th percentile or min/max values as indicated in the figure legend. Histograms and cumulative frequency plots show data pooled from all experiments.

For single molecule experiments, statistics were calculated using python packages scipy and scikit at the per-motor level between experimental groups. The data were tested for normality using D'Agostino and Pearson's test, and as they were not normally distributed, a Kruskal-Wallis test was used to determine if experimental groups differed significantly. Where a statistically significant difference was indicated, pairwise interactions were tested using Conover's post-hoc test and p-values were corrected for multiple comparisons using the Holm-Bonferroni method.

For tail localisation data, statistics were calculated using python packages scipy and scikit at the per-cell level between experimental groups. A Kruskal-Wallis test was used to determine if experimental groups differed significantly. Then, pairwise interactions were tested using Conover's post-hoc test and p-values were corrected for multiple comparisons using the Holm-Bonferroni method.

Cumulative frequency plots were generated using MATLAB and whether distributions are significantly different was tested using Kolmogorov-Smirnov tests. To compare two normal distributions, two sample t-tests were performed.

Data were considered significantly different if $p<0.05$.

Current Biology, Volume *32*

Supplemental Information

Force generation of KIF1C is impaired

by pathogenic mutations

Nida Siddiqui, Daniel Roth, Algirdas Toleikis, Alexander J. Zwetsloot, Robert A. Cross, and Anne Straube

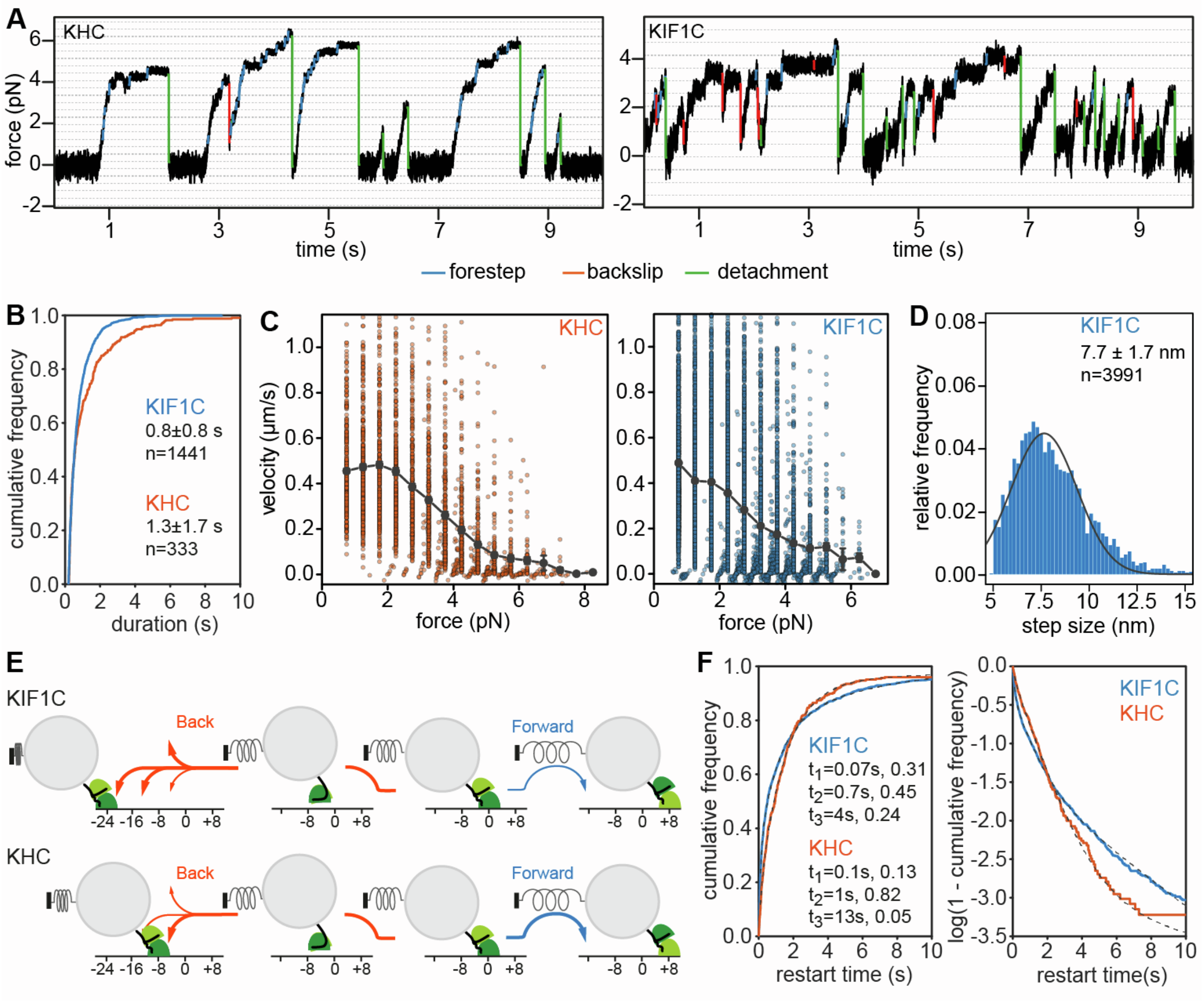

Figure S1: Step and force event analysis, related to Figure 1.
(A) Representative traces from single bead optical trapping experiment for Drosophila melanogaster kinesin heavy chain (KHC) and human KIF1C. Dotted lines indicate 8 nm displacements. The steps detected using the step-finder are highlighted. Forward steps in blue, backslips in red and detachments in green. Parameters used in step finder algorithm are t-test score threshold=30, minimum step size=5 nm, minimum force=1 pN, moving average=20 (1 ms). **(B)** Cumulative distribution of durations over which a force of at least 0.5 pN was maintained, mean ± SD shown, p=0.0002 (Kolmogorov-Smirnov test). **(C)** Distribution of velocity of KHC and KIF1C relative to force. n=353 for KHC and n=1662 for KIF1C. **(D)** Forward step-size distribution for KIF1C. Gaussian curve fit in grey with μ = 7.7 nm, σ= 1.7 nm, n=3991. **(E)** Model highlighting differences in backslipping and restarting probabilities for KIF1C and KHC. KIF1C is more likely to backslip under force and backslips tend to be longer. **(F)** Linear and log plots of cumulative distribution of time lag between force generating events, i.e. time until 0.5 pN force was generated following a backslip or detachment that resulted in force to be reduced below 0.5 pN. n=326 for KHC and n=1404 for KIF1C. Triexponential fits are shown as grey dashed lines and half-times as well as fractions for each mode are indicated in the legend. $p=6 \cdot 10^{-6}$ (Kolmogorov-Smirnov test). See also Methods S1.

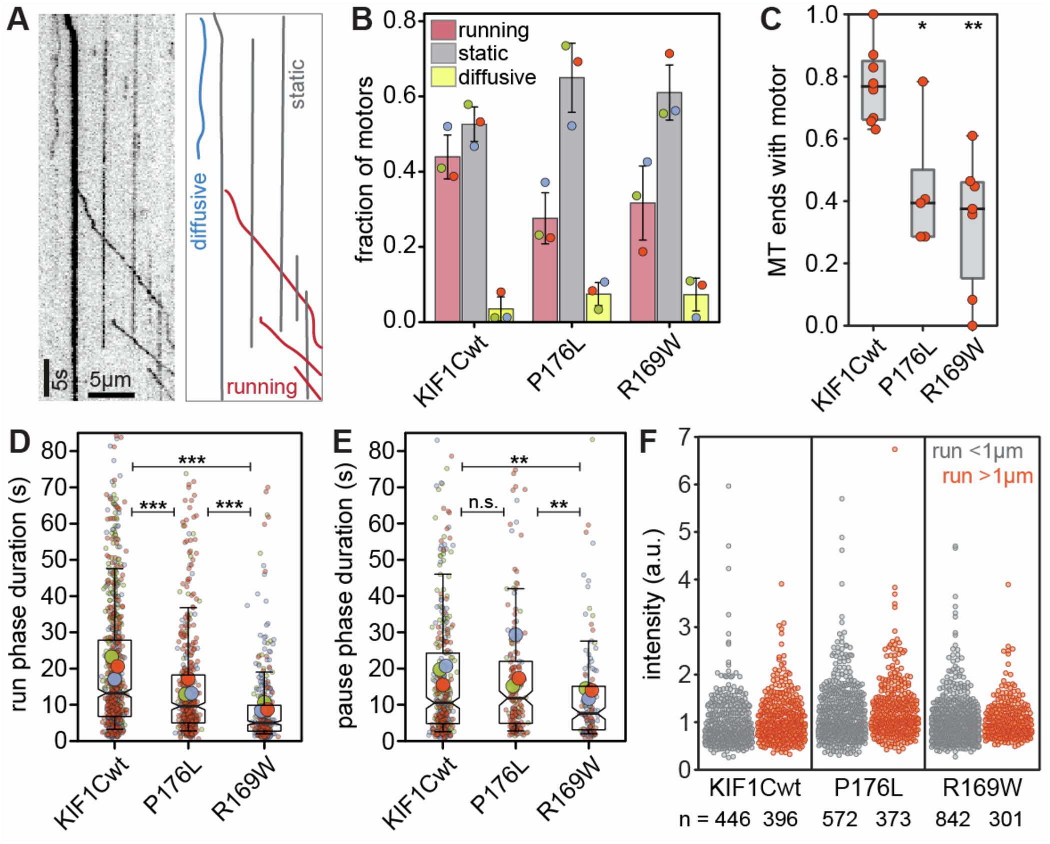

Figure S2: Single molecule behaviour of KIF1C with pathogenic mutations, related to Figure 2.

(A) Three types of behaviour were observed in kymographs: static motors (grey traces), diffusing motors (blue trace) and running motors (red traces). **(B)** Fraction of motors that either run towards the plus end, are static or diffusive. Bars show mean ± SD of 3 experiments, and coloured dots indicate individual values for each experiment. Data from the 3 groups are not statistically different at 0.05 cutoff. (Kruskal Wallis, Conover's test post hoc with Holm correction). **(C)** Fraction of microtubules with at least one motor decorating one end. This was determined at a timepoint about 3 minutes after start of the motility assay, i.e. in the first frame of the second movie in each chamber, by manual assessment. Box plot with data overlay shows median, quartiles and min/max values (whiskers). n = 8, 5, 7 chambers, respectively from 3 independent experiments. * p=0.0164; ** $p=3 \cdot 10^{-4}$ (Kolmogorov-Smirnow test). **(D-E)** Superplots for phase durations for plus end-directed runs and pauses of individual motors (small dots), averages per experiment day (large dots) are shown colour-coded by experiment day. Boxes indicate quartiles and whiskers at $10/90^{th}$ percentiles. Note that values outside of the Y axis limits have been omitted from the graph, but are included in the statistics. n=853, 463 and 384 run phases and 404, 178 and 131 pause phases from 3 experiments. n.s. p=0.52; ** p<0.005; *** p<0.0001 (Kruskal Wallis, Conover's test post hoc with Holm correction). **(F)** Intensity of motors analysed in motility assays separated into a highly processive group with run length > 1 µm (orange) and a group that were either static or underwent a short run < 1 µm (grey). The majority of motors fall into the main peak area of presumably dimers. Higher order motor clusters contribute to a comparable extent to both groups. Number of motors analysed is indicated for each group under the graph. Data were pooled from two independent experiments.
